# In memoriam: Mark Hallett, MD (1943–2025)

**DOI:** 10.1055/s-0046-1827044

**Published:** 2026-08-13

**Authors:** Francisco Cardoso, Joseph Jankovic

**Affiliations:** 1 Universidade Federal de Minas gerais, Departamento de Clínica Médica, Serviço de Neurologia, Unidade de Distúrbios do Movimento, Belo Horizonte MG, Brazil.; 2 Baylor College of Medicine, Department of Neurology, Parkinson's Disease Center and Movement Disorders Clinic, Houston TX, United States.

On November 2, 2025, the world of neurology and clinical neurophysiology lost one of their towering figures: Mark Hallett, MD. He was born in Philadelphia in 1943 and attended medical school at Harvard, where he obtained his medical degree. His lifelong interest in motor control was nurtured by a 2-year fellowship in clinical neurophysiology in the Laboratory of Neurobiology at the National Institute of Mental Health. This was followed by a residency in neurology at the Massachusetts General Hospital. In 1975, he completed a 1-year fellowship at the National Hospital of Neurology (Queen Square, London, United Kingdom), under the supervision of Professor David Marsden. Upon returning to the United States in 1976, he spent 8 years as chief of the Clinical Neurophysiology Laboratory at Brigham and Women's Hospital, in Boston. In 1984, he moved to the National Institute of Neurological Disorders and Stroke (NINDS) at the US National Institutes of Health (NIH). He remained at the NIH until his retirement, in 2022. During the 40 years of affiliation with the NIH, he built a remarkable career as a researcher in movement and motor disorders and, more recently, functional (psychogenic) neurological disorders. With more than 1,200 published scientific papers and an h-index of 185, Dr. Hallett's scientific career is highlighted by the establishment of concepts that have become fundamental parts of contemporary movement disorders and motor neurophysiology. A few examples of those are the understanding of decreased cortical inhibition and the key role of sensory abnormalities in the pathogenesis of dystonia; new classifications of dystonia; the use of botulinum-toxin injections for the management of movement disorders; cortical changes as a potential mechanism mediating the genesis of functional/movement disorders; and many others.

Mark was a man of multiple talents. He was a gifted teacher, capable of crystallizing complex concepts into easy-to-understand principles. It comes as no surprise that one of us (JJ) and Stanley Fahn, one of the founders of the International Parkinson and Movement Disorder Society (MDS) called him *the explainer-in-chief* . He was not an isolated neuroscientist, but he brought his clinical observations from the bedside to his laboratory. His involvement with teaching led him to lecture in essentially all major neurology and neurophysiology meetings in all corners of the globe. This included Brazil, which he visited regularly since 2014. Another facet of his teaching career was the training of more than 100 young physicians and scientists from all over the world. Despite being busy with research and teaching, he still found time to have a strong commitment to administration in institutions outside the NIH. He was president of the MDS, the International Federation of Clinical Neurophysiology, the American Association of Neuromuscular and Electrodiagnostic Medicine, and the Functional Neurological Disorder Society (FNDS), of which he was one of the founders, as well as vice-president of the American Academy of Neurology.

On a personal note, one of us (FC) was a junior member and aspiring leader of the MDS when Mark became its president. Despite the lack of previous personal contact, he was exceedingly supportive during all meetings we attended together in Salzburg, Rome, and other places. Mark was an inspiring leader, able to reconcile his warmth and large smile with rigor in running the business of the MDS as well as that of other organizations with which he was associated as a founder or leader.

**Figure 1:**
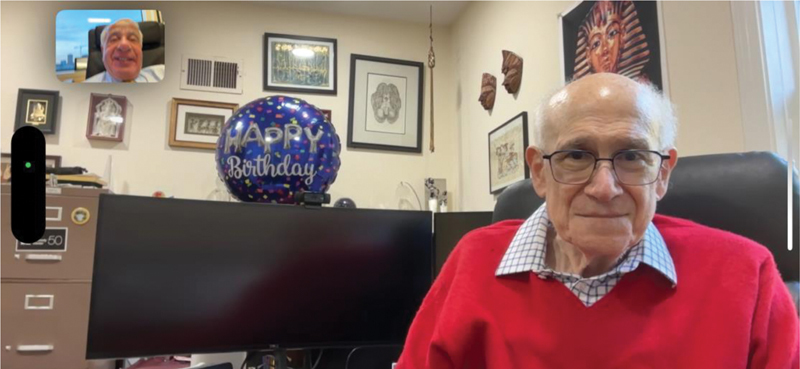
Joseph Jankovic's last (virtual) conversation with Mark, on his birthday (10/22/25).

Dr. Hallett's personal and professional association with one of us (JJ) spans at least 4 decades. In addition to being jointly involved in founding many different societies, including the MDS, the FNDS and the International Neurotoxin Association (INA), we organized numerous international congresses and other meetings. During these frequent interactions, I have always been impressed by Mark's organizational skills and inspiring leadership. More importantly, we became very close friends and frequent travel companions. After the untimely passing of David Marsden, Dr. Hallett joined me and Stan Fahn as a faculty of the “Aspen Course,” now in its 36th year. During the annual Aspen courses, I have had the opportunity to get to know him not only as a colleague but, more importantly, as a warm and generous human being (along with his lovely wife Judy). Through our many light and deep conversations (the last one depicted in Figure 1 ), I have come to fully appreciate Mark's wisdom, encyclopedic knowledge of everything, his engaging humor accompanied by frequent outbursts of infectious laughter, and his deep, deep humanity. He will be missed by us and countless others forever. May his memory be a blessing.

